# The survivability of dialectical behaviour therapy programmes: a mixed methods analysis of barriers and facilitators to implementation within UK healthcare settings

**DOI:** 10.1186/s12888-018-1876-7

**Published:** 2018-09-19

**Authors:** Joanne C. King, Richard Hibbs, Christopher W. N. Saville, Michaela A. Swales

**Affiliations:** 1 School of Psychology, Bangor University, Bangor, Gwynedd UK; 2 Besti Cadwaladr University Health Board, Bangor, Gwynedd UK; 3 Integral Business Support Ltd, Wrexham, UK

**Keywords:** Implementation, DBT, CFIR, Kaplan-Meier, Sustainability

## Abstract

**Background:**

Dialectical Behaviour Therapy (DBT) is an evidence-based intervention that has been included in the National Institute of Health and Care Excellence guidelines as a recommended treatment for Borderline Personality Disorder in the UK. However, implementing and sustaining evidence-based treatments in routine practice can be difficult to achieve. This study compared the survival of early and late adopters of DBT as well as teams trained via different training modes (on-site versus off-site), and explored factors that aided or hindered implementation of DBT into routine healthcare settings.

**Methods:**

A mixed-method approach was used. Kaplan-Meier survival analyses were conducted to quantify and compare survivability as a measure of sustainability between early and late implementers and those trained on- and off-site. An online questionnaire based on the Consolidated Framework for Implementation Research was used to explore barriers and facilitators in implementation. A quantitative content analysis of survey responses was carried out.

**Results:**

Early implementers were significantly less likely to survive than late implementers, although, the effect size was small. DBT teams trained off-site were significantly more likely to survive. The effect size for this difference was large.  An unequal amount of censored data between groups in both analyses means that findings should be considered tentative. Practitioner turnover and financing were the most frequently cited barriers to implementation. Individual characteristics of practitioners and quality of the evidence base were the most commonly reported facilitators to implementation.

**Conclusions:**

A number of common barriers and facilitators to successful implementation of DBT were found among DBT programmes. Location of DBT training may mediate programme survival.

**Electronic supplementary material:**

The online version of this article (10.1186/s12888-018-1876-7) contains supplementary material, which is available to authorized users.

## Background

Dialectical Behaviour Therapy (DBT) [1] is a comprehensive cognitive-behavioural treatment originally developed for adult women who meet criteria for Borderline Personality Disorder (BPD), particularly those who engage in suicidal or non-suicidal self-injury. Traditionally, this client group  has been perceived as “treatment resistant” and considered unsuitable candidates for psychotherapeutic intervention [2]. DBT was the first psychological therapy to challenge the culture of therapeutic rejection for individuals with BPD and has become one of the best evidenced treatments for this client group.

Numerous DBT efficacy trials [3–11] have demonstrated reductions in suicide attempts, intentional self-injury, anger, depression, hopelessness, and improvements in global functioning [12]. Recent meta-analyses have found moderate to large effect sizes indicating a beneficial effect of DBT when compared to treatment as usual on outcomes such as anger, parasuicidality, and mental health [13, 14]. Furthermore, several randomised controlled trials (RCTs) have examined the application of DBT with other client groups such as older adults with major depressive disorder, eating disorders, and forensic populations [15–19]. Thus, the data on DBT clearly indicate its efficacy for the treatment of BPD and holds promise for a host of other disorders.

In 2009, DBT was included in the National Institute of Health and Care Excellence (NICE) guidelines as a recommended treatment for females with a diagnosis of BPD and a history of repetitive self-harm [20]. Since then, a number of healthcare providers within the United Kingdom (UK) have included the provision of DBT as a quality improvement indicator in an effort to meet national targets in health outcomes for individuals with serious mental illness [21]. Preliminary efficiency research also suggests that DBT has the potential to be a cost-effective treatment for individuals presenting with parasuicidal behaviour [22, 23]. Indeed, it appears that the potential benefits DBT has to offer is gaining traction within routine healthcare settings.

Notwithstanding NICE recommendations, demonstrable treatment efficacy, and potential cost efficiencies, concerns have been raised about the sustainability of DBT programmes within the UK National Health Service (NHS) [24]. Diffusion of Innovations Theory [25] suggests that innovations must be widely adopted in order to self-sustain. Widespread adoption of a new practice depends initially on innovators and early adopters and how quickly the subsequent late majority can be persuaded to shift. Furthermore, it is proposed that ideas not sustained by early adopters are unlikely to spread elsewhere [26]. Thus, effective implementation is relevant not only to long-term sustainability but also subsequent spread of an innovation.

Other factors that can impact sustainability are those directly related to the innovation itself, such as the ease with which it can be implemented and how well treatment effects observed in efficacy trials will generalise to routine healthcare settings. The DBT model entails a comprehensive programme that structures the treatment environment across different modalities to enhance client’s capabilities (skills training groups), improve their motivation (individual therapy), aid generalisation of new skills (telephone skills coaching), and supervise DBT therapists (a consultation team model) [27]. All of the treatment modalities are informed by a coherent theoretical model with associated therapeutic strategies based on cognitive behavioural principles and mindfulness [1, 28]. The programme is delivered by a team of mental health professionals all trained within the DBT model and the rationale for doing so is to alleviate the stress and anxiety of working with a high risk client group in which change is often slow [27]. Nevertheless, the requirement of a specialist trained team usually involves significant reorganisation of existing services and an ongoing commitment to delivering an intensive specialist intervention. This is likely to have an impact on how well DBT is implemented or, indeed, whether it is even considered viable for adoption within a service.

Deciding to implement a new practice is not a discrete event but a set of interactive dynamic processes. The difficulties of translating evidence-based research into real-world settings is widely acknowledged [29], which has led to a growing body of literature examining the various factors involved in the implementation and sustainability of evidence-based practices (EBPs) [30–32]. Historically, more attention has been paid to the efficacy of interventions. Whilst such information might help a consumer or agency to select a particular type of intervention, evidence of efficacy alone does not lead to more successful implementation [29], in the same way that simply training practitioners in a new approach does not sufficiently ensure behaviour change [33]. Thus, transfer of innovation needs to be considered within organisational and wider system contexts to ensure that desired change is disseminated, implemented and sustained [34]. However, because organisational restructuring requires changes in service provider behaviour and transformation of systems, translating an EBP into routine practice remains an unquestionably complex and often daunting task.

A number of conceptual frameworks have been developed to aid the process of implementation [29, 31, 35–37]. Whilst these frameworks differ somewhat on areas of emphasis and terminology, influences on implementation generally relate to the context (outer and inner), the innovation itself (fit, training, efficacy), implementation processes (planning, selection, evaluation), individual characteristics (motivation, skill), and sustainability factors (fidelity monitoring, penetration, outcomes etc.). These components are considered to be interrelated and a change in one may result in change to others. Therefore, due to the dynamic nature of healthcare systems and their external contexts, a given programme or practice may require more or less of each component at any one time in order to be successfully implemented. This represents a challenge for the implementation and sustainability of innovations, as the relative contribution of each component to overall outcome can change, resulting in the need for ongoing monitoring of processes. Such tasks can be greatly supported by the application of a guiding theoretical framework. Only recently have distinct models for sustainability of evidence-based programmes been produced [38, 39], however, most of the elements of these models (Inner and Outer Contextual Factors, Characteristics of the Interventionists and of the Intervention) are incorporated already in conceptual frameworks of implementation [32, 36].

Considering the above, implementing a comprehensive DBT programme in routine healthcare settings is unlikely to be a straightforward endeavour. Preliminary research into the sustainability of UK DBT programmes that underwent an intensive training programme between 1995 and 2007 confirmed that some teams had difficulty surviving [27]. Highest failure rates were found shortly after training ended (i.e. the second year of the programme) and again in the fifth year. Participants identified a number of challenges associated with implementing DBT in their service, which were generally characterised by an absence of organisational support. Conversely, for teams that had implemented successfully and managed to sustain, the presence of organisational support was identified as a facilitating factor.

In an effort to increase organisational support and promote effective implementation strategies, British Isles DBT (biDBT) have begun to offer an alternative training modality. Typically, training involves teams of practitioners participating in two five-day DBT intensive training events that are delivered off-site, which is known as the ‘open-enrolment route’. Each training event is separated by 8 months during which teams commence the process of setting up and starting a DBT programme. With the new mode, the content and structure of the training is the same; however organisations wishing to deliver DBT programmes are encouraged to host intensive training on-site. This requires a greater financial investment and consideration of how to adapt staff roles in order to successfully deliver treatment, with the idea that greater organisational investment will have a positive influence on the implementation process. This change in training delivery warrants further investigation to examine whether it improves implementation of programmes.

The aims of the present study are threefold: 1) to investigate whether early and late adopters of DBT have differential sustainability, 2) to investigate whether change in training method delivery impacts the sustainability of DBT programmes, and 3) to examine factors that act as a barrier or facilitator to implementation by using a theoretical implementation framework to guide assessment.

## Method

### Participants

All biDBT programmes that underwent Intensive Training™ between January 1995 and February 2016 were eligible for this study. During this period, whether at on-site or off-site trainings, both the structure and content of the DBT Intensive Training™ remained constant, with only minor modifications to the order of topics taught. All trainings were delivered by two or three members of a six person team who had all been trained to a consistent standard of training, all of whom were adherent DBT therapists. For the sustainability analyses, the unit of analysis was DBT teams. For the survey arm of this study, only one team member from each DBT programme was invited to participate in the study. In the first instance, all DBT team leaders were invited to participate. If a team leader was unavailable, another current team member of an active team, or any former member of inactive teams, was invited to participate.

### Design & Procedure

A concurrent mixed-methods approach was employed [40]. Sustainability of DBT programmes was quantified using Kaplan Meier (K-M) [41] survival analysis. biDBT maintain a database to systematically record data on programme start date, activity status (i.e. active or inactive programme), cessation date, and site of training delivery. During the period of the study all programmes were contacted by telephone to establish if they were still active i.e. delivering a DBT programme to clients, consistent with one of Scheirer’s [42] definitions of sustainability. These data were used to analyse survival rates as a proxy for sustainability.

Survival data were triangulated with responses from an online survey to identify factors that may aid or hinder implementation of DBT in routine settings. Initial contact to participate in the survey was made via email to all DBT team leaders registered on the biDBT training database. If an email was returned as undeliverable, an alternative team member was contacted. Participants were provided with information on the purpose of the study and were offered the opportunity to be entered into a prize draw following completion of the survey. A link to the online survey was contained within the body of the initial email.

### Measures

A 70-item online questionnaire (Additional file 1) was designed to elicit information regarding DBT teams’ experiences of implementing DBT in their service. The questionnaire consisted of three types of questions (closed, free response, and rating scales) and was conceptually divided into six separate domains. The first domain relates to factors considered to be relevant to practice sustainability and is adapted from Swain and colleagues’ [43] study on the sustainability of EBPs in routine mental health agencies. The remaining five domains are based on Damschroder and colleagues’ [36] Consolidated Framework for Implementation Research (CFIR). The CFIR is an overarching theoretical framework that incorporates common constructs from a range of published theories on implementation and is comprised of five major domains*: Intervention Characteristics; Inner Setting; Outer Setting; Individual Characteristics*; and *Implementation Processes.* Each domain includes a constellation of interactive constructs that are purported to influence the implementation process, for a detailed discussion see [36]. Demographic information was also collected.

### Analysis

Kaplan-Meier (K-M) [41] survival analyses were carried out to estimate the cumulative survival rates of DBT programmes. Based on the biDBT database teams were ascertained as either *active or inactive.* Teams that could not be contacted were considered lost to follow-up. Whilst including teams lost to follow-up as censored data is standard practice in K-M analysis, the analysis makes no distinctions within the censored data between teams that cannot be contacted (i.e. lost to follow up) and those that are still functioning. Including teams lost to follow-up as censored (i.e. assuming they are still alive) may make the survival estimate unreliable, we therefore excluded them from the survival analyses.

#### Study aim 1

To investigate whether there were differences in sustainability between early and late adopters, a K-M analysis comparing survival rates of teams trained between January 1995 and March 2007 (12 years) with teams trained between April 2007 and February 2016 (9 years) was carried out (*N* = 468). Programme start and cessation dates were used to calculate survival rate. To reduce the potential for unequal amounts of censored data between groups due to differences in duration of cohort timeframes (12 versus 9 years), only the first seven years of a programme within these time frames were analysed. Programmes that survived for at least 2555 days were censored regardless of whether they later became inactive. Teams active at the time of analysis (or active for at least 2555 days) were categorised as censored data. A chi-squared test was used to check for differences in the amount of censored data between groups. A log-rank test was used to test whether the rate of programme closure varied between groups. A Cox regression model was also fitted to estimate a hazard ratio between groups, as log-rank analyses do not yield effect sizes.

#### Study aim 2

To examine whether training method delivery influenced the sustainability of DBT programmes, a K-M analysis comparing teams trained on-site with teams trained via open-enrolment was carried out. Teams were allocated to their respective study group based on site of training delivery. This information was extracted from biDBT database. Survival rates were calculated using programme start and cessation date. Programmes active at the time of analysis were categorised as censored data. Only DBT programmes that commenced training from January 2009, the date at which the off-site training model was introduced were included in this analysis. A chi-squared test was used to check for differences in the amount of censored data between groups. A log-rank test was used to test whether the rate of programme closure varied between training methods. A Cox regression model was also fitted to estimate a hazard ratio between groups, as log-rank analyses do not yield effect sizes.

#### Study aim 3

A descriptive content analysis of survey data was carried out by the first author to investigate the frequency with which individual implementation and sustainability constructs were identified as an aid or barrier to a programme’s ability to successfully implement and sustain.

## Results

### Survival analyses

#### Study aim 1: Early versus late cohort comparison

**Fig. 1:**
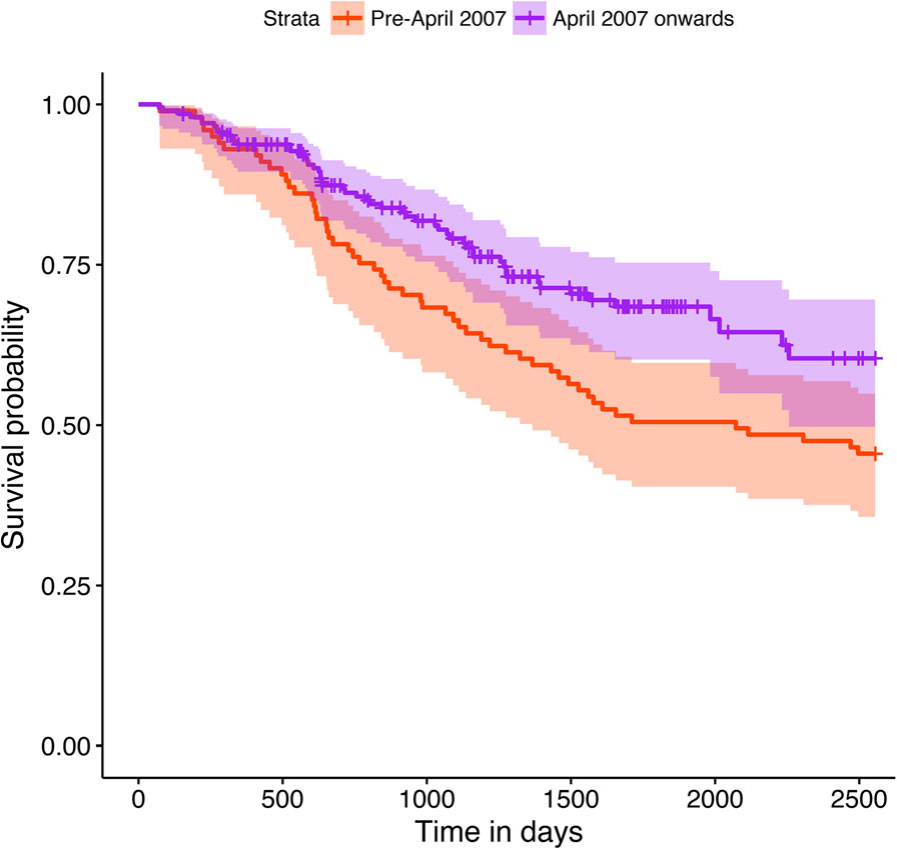
Comparison of survival curves between DBT programmes trained prior to and post April 2007

A total of 468 teams were included for analysis. Of these, 160 teams were from the pre-April 2007 cohort (inactive *n* = 55, active *n* = 46) and 308 teams (inactive *n* = 157, active *n* = 55) were from the post-April 2007 cohort. A chi-squared test indicated significant differences in the distribution of active, and inactive teams between the pre and post April 2007 groups (χ^2^ = .23.164, df = 1, *p*-value = 1.488e-06), in that the post-April 2007 group had more censored and less inactive data than the pre-April 2007 group. K-M survival curves (Fig. 1) and log-rank test indicated that the pre-April 2007 group had a faster rate of closure than the post-April 2007 group (χ^2^ = 6.819, *p* = .009). Cox regression indicated that the hazard ratio was 0.607 (95% CI = 0.416–0.886, reference category = pre-April 2007 group) with a Cohen’s d approximation = −.389. Highest programme failure rates were found in the second year for both cohorts.

#### Study aim 2: Training method comparison

**Fig. 2:**
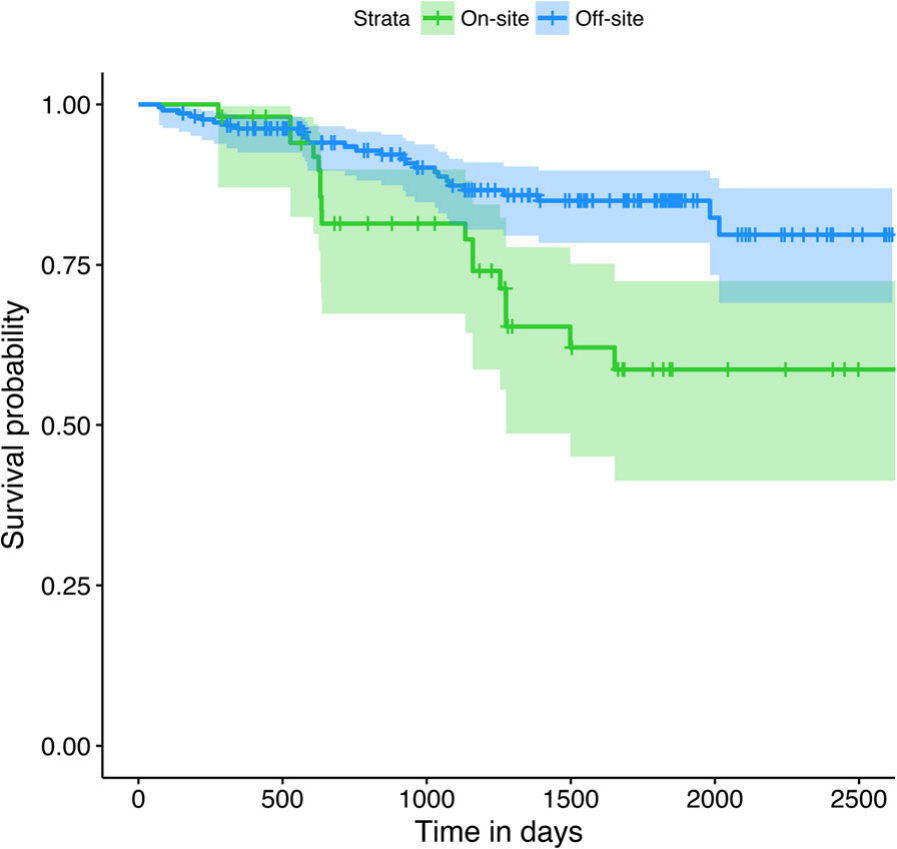
Comparison of survival curves between DBT programmes trained off-site and onsite

A total of 266 teams were included for analysis. Fifty-two teams (active *n* = 35, inactive *n* = 17) were trained on-site and 214 teams (active *n* = 187, inactive *n* = 27) were trained off-site. A chi-squared test indicated greater levels of censored data in the on-site group (χ^2^ = 10.802, *p* = .001). K-M survival curves (Fig. 2) and log rank test showed that teams trained off-site had a significantly higher probability of survival than teams trained on-site (χ^2^ = *9.*801, *p* = .002). Cox regression indicated that the hazard ratio was 2.554 (95% CI = 1.392–4.688, reference category = off-site) with a Cohen’s d approximation = 0.731). Highest failure rates were found in the second year for teams that trained on-site, compared to the third year for teams trained via open-enrolment.

### Barriers and facilitators to implementation

#### Study aim 3

The online questionnaire was completed by 68 respondents. Sixty-two (91%) were from active teams and 6 (9%) were inactive. Of the active teams, the majority of respondents were located in England (*n* = 38, 61%) and the remainder were located in Wales (*n* = 8, 13%), Scotland (*n* = 2, 3%), and Ireland (*n* = 8, 13%). The proportion of teams containing the following professions were: clinical psychologists (*n* = 56, 83%), nurses (*n* = 52, 77%), social workers (*n* = 22, 33%), psychological therapists (*n* = 22, 33%), and occupational therapists (*n* = 13, 21%). The most frequently reported number of DBT trained clinicians within a service was between 4 and 5 (*n* = 23, 37%), with a range of 2 to 12 trained clinicians. Twenty-nine (46%) respondents worked within community adult mental health services, 12 (19%) within child and adolescent mental health services (CAMHS), and the remainder across a range of learning disability (*n* = 3, 5%), eating disorders (*n* = 2, 3%), forensic (*n* = 7, 10%), youth mental health (*n* = 1, 2%), personality disorder (*n* = 1, 2%) and inpatient settings (*n* = 9, 13%). Fifty-three (85%) active teams fell within the statutory service sector and 9 (15%) within the private sector.

Of the six inactive teams who completed the online survey, the median survival time was 2015 days (5.5 years), range 635–4405 days. All respondents from inactive teams were asked to provide three reasons why they thought their DBT programme discontinued. The most frequently cited reason for programme failure was lack of management support (*n* = 5, 83%) either due to lack of understanding of how DBT works, insufficient time allocated to deliver DBT, or priority given to competing service demands. Lack of funding (*n* = 3, 50%), lack of colleague support (*n* = 3, 50%), and staff turnover (*n* = 2, 33%) were other reasons reported for programme failure. One respondent also cited high dropout rates as a reason for their programme ending but reflected that this may have been as a result of “overly rigid referral criteria”.

### Content analysis

Response frequencies and percentages for each implementation construct were counted for the total online survey sample. Respondents were also invited to leave comments to further elaborate their responses within each implementation domain. All comments were analysed by the lead author and grouped according to the implementation category referred. Due to the small response rate from inactive teams, comparative analyses of response differences between active and inactive programmes could not be carried out.

### Barriers to implementation

The most frequently endorsed barrier to implementing DBT was practitioner turnover (*n* = 40, 59%) followed closely by financing (*n* = 35, 52%). Other common barriers were availability of resources (*n* = 28, 41%), the perceived difficulty of implementing DBT (*n* = 27, 40%), and external change events (*n* = 23, 34%). No constructs within the *Individual Characteristics* or *Outer Setting* domains were strongly endorsed as barriers to implementation. Table 1 provides illustrative comments to the most commonly reported barriers to implementing DBT.

**Table 1:** Barriers to Implementing DBT

Implementation domain	Construct	*N*	%	Example
Intervention characteristics	Financing Perceived difficulty of implementing DBT	35 29	52 40	“Cost of DBT training can be prohibitive…concern about this in future in current economic climate - despite evidence base for longer term money saving - trusts often view things in short term when lots monies need to be saved” “All DBT staff have had a long break since last running the programme and so it is harder for us to re-start the programme”
Inner setting	Practitioner turnover Available resources	40 28	59 41	“Until very recently we had no practitioner turnover this really helped with the initial establishment of DBT and refining it. We have recently had someone leave and one person is on mat leave…The people who have left are our least psychologically experienced team members and so these people delivered the groups whilst others did more primary therapy. At the moment existing team members are now doing both and this is not sustainable long term.” “Failure to provide funding for a second laptop for second consecutive group and time in lieu for out-of-hours telephone consult hindered implementation.”
Implementation process	External change events	23	34	–

*Note.* - indicates no elaborative comments provided for implementation construct

### Aids to implementation

There were a number of constructs strongly endorsed as aiding the implementation process, the most common being the quality of the DBT evidence base (*n* = 60, 88%). Other frequently endorsed constructs were practitioner skills (*n* = 56, 82%), acceptability of DBT by clients (*n* = 54, 79%), the perceived advantage to implementing DBT into practice (*n* = 53, 78%), practitioner attitudes (*n* = 53, 78%), DBT training (*n* = 52, 77%), practitioner readiness (*n* = 51, 75%), and shared willingness among DBT clinicians to implement the programme (*n* = 51, 75%). All constructs within the *Individual Characteristics* domain were strongly endorsed as aiding the implementation process. Illustrative comments are provided in Table 2.

**Table 2:** Aids to Implementing DBT

Implementation domain	Construct	*n*	%	Example
Intervention characteristics	Quality of DBT evidence base Perceived advantage of implementing DBT DBT training	60 53 52	88 78 77	“Evidence on efficacy and cost savings also had a significant impact in securing Trust manager’s interest and support” “Business plan presented to commissioners comparing costs of often unsuccessful inpatient programmes, allegedly DBT informed, with adherent programme.” “The training we had from the British Isles team was excellent and central to our success. We make reference to it frequently in consult meetings.”
Outer setting	Acceptability of DBT by clients	54	79	“In the past, when DBT was at risk of cuts due to financial pressures, we were able to arrange for ex-clients and current clients to talk to the senior management and explain the impact and benefits DBT had had on their lives.”
Inner setting	Shared willingness to implement DBT Leadership engagement	51 49	75 72	“We regularly meet for CPD opportunities (every 6 months) on DBT adherence and how we are implementing DBT. We use recordings/triadic observation of the 1:1 session to evaluate therapist behaviours and try to stay focused on the Consultation Supervision group agreements.” “…so there is senior management support to find a solution quickly. Including to find resource to train a considerable number of new staff and ensure that their roles in relation to DBT are made clear going forward.”
Individual characteristics	Practitioner skills Practitioner attitudes Practitioner readiness	56 53 51	82 78 75	“Clinicians highly skilled and experienced so take great pleasure in learning and adhering to effective but also very creative model.” “We have a team of highly motivated DBT therapists and the service has developed a good and growing reputation with referrers to the service.” -
Implementation process	Appointment of DBT team leader Execution of implementation plan	42 42	62 62	“…but the DBT lead worked to gain this [management buy-in] and the success of the programme has led to this over time.” -

*Note.* - indicates no elaborative comments provided for implementation construct

### Sustainability

Frequency and percentage data were collected on a number of factors considered to be related to sustainability of interventions such as collection of client outcome data, extent of programme penetration, ongoing training and consultation, and treatment fidelity. Of the active teams, 51 (82%) collected client outcome data, which was mainly used for tracking client progress and auditing the effectiveness of the programme. Seven (11%) respondents indicated that they were serving considerably fewer clients than when they initially commenced DBT training. Twenty-nine teams reported that they were serving approximately the same (47%) and 26 (42%) said they were serving a lot more clients since initial training. Thirty-seven (60%) respondents had received external consultation. However, only 24 (39%) reported accessing DBT expert supervision. The majority of teams, 43 (69%), carried out new team member training and 34 (55%) had received booster training. With regards to treatment modalities, 61 teams (98%) offered skills training and individual therapy, 60 (96%) ran a consultation group, and 48 (77%) offered telephone support. Finally, 41 teams (66%) had made adaptations to the DBT model and of these, 20 (32%) reported making changes during the initial training phase.

All six inactive teams collected outcome data. Four teams used the data (67%) to demonstrate clinical outcomes and cost effectiveness. One respondent (17%) indicated that they had served considerably fewer clients post initial training phase, with the remaining respondents either having served the same amount (*n* = 2, 33%) or a lot more clients (*n* = 3, 50%). Only two teams (33%) did booster training and no teams carried out new team member training. Five teams (83%) had offered all four DBT treatment modalities: individual therapy, group skills training, therapist’s consultation group, and 24-h telephone access. One team (17%) did not offer telephone consultation. Only two teams (33%) reported modifying the DBT model to suit their service needs and of these, one team made modifications during the initial training period whilst the other implemented one full round of DBT before making adaptations.

## Discussion

Consistent with earlier data [27], the highest failure rate for DBT programmes was observed in the second year post-training. Despite this early high failure rate the survivability of DBT programmes compares well with other evidence-based programmes reported in the literature. Cooper and colleagues [44] reported that 69% of delinquency and violence prevention programmes in a state-wide implementation sustained at two to four years post-initial seed-funding. Whilst the National Implementing Evidence Based Practices project reported that 80% of sites sustained at two years post implementation [45] and 47% at six years, although, in the six year data, sustainability rates varied between the five interventions studied from 25 to 69%. DBT compares favourably with these figures with survivability rates of 88% at two years and 69% at eight years.

Differences in the survival rates between the early and late implementers is not particularly surprising, although the different rates of censored data between the cohorts means that the result should be interpreted with caution. Several factors might account for this difference. Early adopters are known to be psychologically different from their peers and often in influential positions [46]. Whilst they may have adopted DBT early they may also have been keen to move on to the next innovation. Secondly, DBTs place as an evidence-based intervention within the UK became more solid with the publication of the NICE guidance in 2009 [20]. The advocacy for DBT within the guideline may have provided an ‘outer context’ support to teams training post-2007, just as publication of the guidance also boosted training in DBT [47].

Traditionally, the translation from science to practice has been a passive process that has usually only involved diffusion and dissemination of EBP information, with the hope that this is sufficient to change practitioner behaviour. There is a current shift towards a more active approach whereby outside experts work alongside organisations to help achieve implementation success and assure benefits to consumers [48]. Results from the present study found that on-site training did not increase the probability of survival. Survival curve comparison of training delivery methods indicated programmes trained off-site had a significantly higher probability of surviving. This is a surprising finding, given that on-site training was designed to increase organisational investment in DBT implementation. However, this finding must be interpreted with caution, as the amount of censored data between the comparison groups was found to be significantly different, limiting conclusions that can be drawn about differences between groups. Notwithstanding this caveat, a possible explanation for the differences may be that those attending off-site training have engaged in a substantial amount of pre-planning and assessment of organisational readiness, and in efforts to obtain management buy-in, have identified an explicit need for implementing DBT in their service setting. In doing so, they are possibly more likely to have actively considered how an implementation plan may be executed. Addressing organizational funding and resources and aligning the innovation with organizational goals are factors known to be associated with sustainability [39, 43, 45, 49]. Teams attending off-site training have typically had to actively pursue funding and gain agreements from their organisation to attend. This may indicate that individuals in teams pursuing this route may possess particular leadership skills that may also relate to sustainability [49, 50]. Attending off-site training provides greater opportunities to network with other teams, allowing for the sharing of experiences and ideas, which prove beneficial to implementation and sustainability. During the second week of training teams present their initial implementation efforts for consultation and feedback from trainers and fellow trainees. In off-site trainings trainees are exposed to a wider range of systems and witness trainers applying the components of the model to these different systems. This more expansive experience may increase knowledge of the core components and principles of DBT. Cooper and colleagues [44] similarly highlighted that greater knowledge of a progammes’ logic model increased the likelihood of sustainability.

Practitioner turnover and financing were the most commonly identified barriers to implementing DBT programmes. This is consistent with findings from other studies [43, 45, 50]. Indeed, these constructs may interact, as difficulty financing new team members was one of the main problems identified when practitioner turnover was high. Financing initial training was identified as a key barrier for some programmes. Although, a few overcame this difficulty by securing initial funding from external sources and then using evaluation and outcome data to secure ongoing funding from their organisations. Other programmes identified difficulties with ongoing financing, whether it was for training new team members, booster training, or accessing expert supervision or consultation. Whilst securing financing is a common theme both in this study and in others [43, 45, 50] consideration is rarely given to the costs of de-implementation and, in the case of DBT, failing to provide an intervention that may produce cost-savings [22]. Developing models that highlight the costs of failing to sustain may prove useful to influence leaders both in the inner and outer context or organisations to continue to support an evidence-based intervention.

A number of facilitators to implementation were identified. Most notably, all constructs within the *Individual Characteristics* domain were strongly endorsed as aiding the implementation process. A number of respondents reported highly motivated or skilled practitioners, effective leadership of the DBT team, or the presence of a DBT champion as key to overcoming barriers encountered to implementation and sustainability of programmes. This finding highlights how a strength in one or more areas can compensate for weaknesses in others [29]. Nevertheless, overreliance on an individual(s) to ensure effective implementation and sustainability leaves a programme particularly vulnerable to practitioner or leadership turnover. Organisations are dynamic and so the relative contribution of implementation constructs can inevitably wax and wane. This poses a difficulty for organisations because changes in one construct requires adjustments in others. Thus, successfully managing such changes will require effective monitoring and feedback systems to keep a programme on track [48], as well as ongoing availability of resources to do so.

Characteristics of the intervention, a feature in many implementation and sustainability models [31, 36, 38, 39] in particular the quality of the evidence base for DBT, was strongly endorsed as aiding the implementation process. Whilst efficacy data alone maybe insufficient for changing practice, findings from this study indicated that for some programmes research data played a crucial role in securing management commitment to delivering DBT. The quality of the evidence base may be of particular relevance during pre-planning and preparation stages, allowing for organisations to weigh up the suitability of DBT for their service and make as assessment of perceived benefits and ‘fit’ with the context [38]. For populations where the evidence base for DBT is not as extensive or robust, the lack of efficacy data may present a barrier to implementation. In this instance, the opportunity to trial a DBT programme and collect effectiveness data may prove beneficial.

Over half of survey respondents indicated that their programme engaged in practices which are considered pertinent to sustainability, with the exception of receiving supervision from a DBT expert. This is an encouraging finding and suggests that teams are aware of the need for continuous monitoring and collection of outcome data as an aid to sustainability [43]. Given that the highest failure rates for programmes are found within the active implementation stage (i.e. first two years), programmes should also consider identifying and monitoring implementation outcomes, distinct from service and treatment outcomes. Evaluation of implementation outcomes will provide an indicator of implementation success and yield an index of implementation processes. Also, because treatment effectiveness requires successful implementation, monitoring implementation outcomes is a necessary intermediate step to obtaining desired clinical and service outcomes [51].

There are a number of limitations to the study. The first being the small number of survey respondents from inactive teams, which prevented comparative analyses, and limits the conclusions that can be drawn from the findings. Second, the method of data collection prevented exploration of research participants’ interpretation of questions or the opportunity to clarify responses. Although a summary question was included at the end of each survey domain, not all respondents chose to elaborate their responses, limiting the amount of qualitative data collected. Lastly, the retrospective accounts from individual team leaders/members must be interpreted with caution due to problems inherent with self-report, such as post-hoc rationalisation. Future research should endeavour to recruit multiple respondents from programmes to reduce the likelihood of methodological bias, as well as recruit greater numbers of inactive teams to ensure a representative sample of respondents.

Despite these limitations, the present study possessed a number of strengths. There are few studies in the literature studying sustainability beyond the early stages of implementation (post-two years) and none, to our knowledge, that allow the comparison of two different types of training delivery that may have implications for sustainability. In addition, the use of a concurrent mixed-methods approach allowed quantitative findings to be complimented with qualitative information providing greater insight into the complexities of implementation and sustainability processes. The existing implementation literature utilizes a wide range of definitions and terminologies rendering extrapolation of constructs across settings difficult. By using the CFIR as a scoping tool to guide assessment of the barriers and facilitators to DBT, a number of constructs salient to implementing DBT in routine healthcare settings were identified, allowing for refinement of more relevant assessment tools for future research.

## Conclusions

Successful implementation and sustainability of healthcare innovations into routine settings poses a challenge; DBT is no exception. However, since the onset of biDBT intensive training in 1995, the sustainability of DBT programmes has remained stable and similar to the rates of other innovations, and higher than others. Given the ever-changing landscape and finite resources of healthcare systems, this is an encouraging finding. Nevertheless, a number of programmes struggle to effectively implement and sustain DBT within their organisation. The particular adaptation to the location of training trialed here did not improve the probability of programme survival. Further augmenting on-site training with additional interventions for both inner and outer-context leadership [49, 50] could potentially improve the outcome of such training . A number of factors hindering or facilitating implementation of DBT were reported. Whilst these factors can vary between and within organisations, comparison with previous research suggests that the main barriers or aids to implementation have remained fairly consistent. Future research should include evaluation of predictive models that allow for testing the relative contribution of each implementation component, in order to identify what works in which contexts.

## Additional file

Additional file 1: Online Survey Questionnaire. (PDF 10203 kb)

## Abbreviations

biDBT: British Isles Dialectical Behaviour Therapy training team
BPD: Borderline Personality Disorder
CAMHS: Child and Adolescent Mental Health Service
CFIR: Consolidated Framework for Implementation Research
DBT: Dialectical Behaviour Therapy
EBP: Evidence Based Practice
K-M: Kaplan Meier survival analysis
NHS: National Health Service
NICE: National Institute for Health and Care Excellence
RCT: Randomised Controlled Trial
UK: United Kingdom
