# Supplementary material for: The survivability of dialectical behaviour therapy programmes: a mixed methods analysis of barriers and facilitators to implementation within UK healthcare settings
Source: BMC Psychiatry. 2018 Sep 19;18:302. doi: 10.1186/s12888-018-1876-7 (PMC6146662; doi:10.1186/s12888-018-1876-7)
Supplement: Supplementary file 1 — Online Survey Questionnaire. (PDF 10203 kb) [file 12888_2018_1876_MOESM1_ESM.pdf]

DBT Implementation Survey

Page 1

Welcome to the DBT Implementation Survey.

This research aims to examine the factors related to the successful implementation and sustainment of DBT porgrammes within routine UK and Ireland healthcare settings. By examining the implementation process, we hope to gain valuable information regarding which circumstances are most likely to lead to programme success or programme failure.

The questionnaire has been devised based on the Consolidated Framework for Implementation Research (CFIR, Damschroder et al., 2009). Implementation is a complex process and has been conceptually divided into the following domains: intervention characteristics, outer and inner setting, characteristics of individuals, implementation processes, and sustainability. Some of the questionnaire items relating to each domain may be more relevant to your experience than others. However, any items that you deem non-relevant are of equal interest in our examination of the factors that aid or hinder implementation. Therefore, we would welcome any explanation as to why you may deem a particular item relevant or non-relevant.

There are three types of questions within the questionnaire: some are simple factual questions, others should be answered in your own words, and the last type are questions answered on a rating scale.

In submitting responses to this survey, the participant consents to take part and recognises that the information provided will be used for the purposes of the current study. All responses will be confidential and all published results will be anonymised.

You may stop participating in the research at any time. Should you wish to withdraw following submission of your responses, please contact the principal investigator named on the information sheet, and your data will be removed from the study and destroyed.

Section A

Are you still offering DBT?

- Yes
- No

If no, go to Section B by hitting next

When did you start to offer DBT in your service (round up to the nearest month and year)?

How many DBT clinicians are there in your service?

How many whole time equivalents (WTE) are in your DBT team? If you are unsure of this figure, please provide the sum total of the number of days each clinician devotes to DBT.

What is the professional categorisation of the DBT clinicians in your service?

- Clinical Psychologist
- Social Worker
- Nurse
- Psychological Therapist
- Counsellor
- Other

If you selected Other, please specify:

What is the location of your service?

- England
- Wales
- Scotland
- Northern Ireland
- Ireland

Was your team trained:

- On service site
- Off service site

Please state the nature of your service (e.g. AMH, CAMHS, etc.)

Which sector does your service fall under?

- Statutory
- Private

Section B

When did you stop offering DBT (please enter response in mm/yyyy format) * Required

Please tell me 3 things, in or out of your control that you think worked against sustaining DBT in your service. That is, please tell me why you think your service no longer offers DBT. * Required

Section C

Outcomes

Are you measuring client outcomes related to DBT?

☐ Yes ☐ No

If yes, how are the outcome data used?

Who sees the data?

How often and how long after the time period covered?

Penetration

How many clients are you serving with DBT now compared to when training had just been completed?

- considerably less
- about the same
- a lot more

Training/Consultation

Do you do new team member training?

- Yes
- No

Do you do booster training?

- Yes
- No

Have you sought advice concerning DBT from outside consultants within the last two years?

- Yes
- No

How much external consultation have you had in the last two years (i.e. DBT expert comes on-site to visit team)?

Have you had supervision from a DBT expert (i.e. weekly session review of therapy tapes typically by phone/in-person)?

- Yes
- No

Fidelity

Which aspects of DBT do you offer (please tick all that apply)

- One-to-one
- Skills training
- Consultation group
- Telephone support

How frequently and for how long each week does your consultation team meet?

Have you modified the DBT model to suit your service needs? That is, have you made changes to DBT in order to adapt to such things as socio-cultural milieu, local regulations or policies, client characteristics, practitioner skills or experience, or recent

research findings?

- Yes
- No

If yes, please describe briefly the local adaptations to the DBT model?

To what extent have you adapted DBT? Please rate the extent of the adaptations on a scale of 1 to 5, with 1 indicating a little and 5 indicating considerable adaptations.

	1	2	3	4	5	
Little adaptation						Considerable adaptation

At what stage in the implementation process did you make the adaptations?

- During initial training
- Once training was completed and 1 or more attempts of adhering to the DBT model had occurred

Is there anything else you would like to add to help in our understanding of the sustainment of DBT in your service?

Section D

Outcomes

Did you measure client outcomes related to DBT?

- Yes
- No

If yes, how were the outcome data used?

Who saw the data?

How often and how long after the time period the data covered?

Penetration

Following the initial training period, how many clients were you serving with DBT?

- considerably fewer than when training
- about the same
- a lot more

Training/Consultation

Did you do new team member training?

- Yes
- No

Did you do booster training?

- Yes
- No

Did you seek advice concerning DBT from outside consultants whilst your DBT programme was active?

- Yes
- No

How much external consultation did you have when your programme was active?

Did you receive supervision from a DBT expert (i.e. weekly session review of therapy tapes typically by phone/in-person)

- Yes
- No

Fidelity

What aspects of DBT did you offer (please tick all that apply)?

- One-to-one
- Skills training
- Consultation group
- Telephone support

How frequently and for how long each week did your consultation team meet? Please enter 0 if you did not carry out this aspect of DBT

Did you modify the DBT model to suit your service needs? That is, did you make changes to DBT in order to adapt to such things as socio-cultural milieu, local regulations

or policies, client characteristics, practitioner skills or experience, or new research findings?

- Yes
- No

If yes, please describe briefly the local adaptations you made to the DBT model.

To what extent did you adapt DBT? Please rate the extent of the adaptations on a scale of 1 to 5, with 1 indicating a little and 5 indicatiing considerable adaptation.

Little adaptation ☐ ☐ ☐ ☐ ☐ Considerable adaptation

At what stage in the implementation process did you make the adaptations?

- During initial training
- Once training was completed and 1 or more attempts of adhereing to the DBT model had occurred

Is there anything else you would like to add that would help our understanding of why it was difficult to sustain DBT within your service?

Section E

The following are factors that may affect implementation of evidence-based practices. For each one, please choose on a scale that best describes its impact on your service's ability to implement DBT. The scale ranges from -2 to +2. A negative number indicates a factor that worked against successfully implementing DBT. A positive number indicates a factor that worked towards implementing DBT. The midpoint of the scale (0) indicates that the factor had no effect or that the negative and positive effects cancelled each other out.

Intervention Characteristics

Was the source of the decision to implement DBT in your service internal or external?

- Internal
- External

In what way, if any, did this affect implementation?

	-2	-1	0	1	2	
Hindered our attempts to implement successfully						Helped us to implement successfully

Quality of the evidence base for DBT

	-2	-1	0	1	2	

Hindered our attempts to implement successfully	☐	☐	☐	☐	☐	Helped us to implement successfully

Perception of the advantages of implementing DBT in your service

	-2	-1	0	1	2	
Hindered our attempts to implement successfully	☐	☐	☐	☐	☐	Helped us to implement successfully

Extent to which DBT can be tailored to meet the needs of your service

	-2	-1	0	1	2	
Hindered our attempts to implement successfully	☐	☐	☐	☐	☐	Helped us to implement successfully

Trialability (i.e. the ease in which DBT could be piloted in your service before implementation)

	Strongly disagree	Disagree	Neither agree nor disagree	Agree	Strongly Agree

Hindered our attempts to implement successfully	☐	☐	☐	☐	☐	Helped us to implement successfully

Perceived difficulty of implementing DBT within your service

	-2	-1	0	1	2	
Hindered our attempts to implement successfully	☐	☐	☐	☐	☐	Helped us to implement successfully

DBT training

	-2	-1	0	1	2	
Hindered our attempts to implement successfully	☐	☐	☐	☐	☐	Helped us to implement successfully

Financing of DBT

	-2	-1	0	1	2	
Hindered our attempts to implement successfully	☐	☐	☐	☐	☐	Helped us to implement successfully

Would you like to expand further on any of the responses you have provided on this page?

Outer Setting (this includes the economic, political, and social context in which your service resides)

Involvement of clients and families in DBT

	-2	-1	0	1	2	
Hindered our attempts to implement successfully	☐	☐	☐	☐	☐	Helped us to implement successfully

Acceptability of DBT by clients

	-2	-1	0	1	2	
Hindered our attempts to implement successfully	☐	☐	☐	☐	☐	Helped us to implement successfully

Accessibility of DBT for clients

	-2	-1	0	1	2	
Hindered our attempts to implement successfully	☐	☐	☐	☐	☐	Helped us to implement successfully

Consultation with external agencies

	-2	-1	0	1	2	
Hindered our attempts to implement successfully	☐	☐	☐	☐	☐	Helped us to implement successfully

Have you received external supervision?

- ○ Yes
- ○ No

What impact, if any, did this have on implementation of DBT? *
Required

	-2	-1	0	1	2	
Hindered our attempts to implement successfully	☐	☐	☐	☐	☐	Helped us to implement successfully

Competitive pressure with other services/agencies

	-2	-1	0	1	2	
Hindered our attempts to implement successfully	☐	☐	☐	☐	☐	Helped us to implement successfully

Government or local health board policy

	-2	-1	0	1	2	
Hindered our attempts to implement successfully	☐	☐	☐	☐	☐	Helped us to implement successfully

Would you like to expand further on any of the responses you have provided on this page?

Inner Setting (includes the structural, communication, and cultural characteristics of your service)

Social architecture of service (e.g. age, size, level of expertise)

	-2	-1	0	1	2	
Hindered our attempts to implement successfully	☐	☐	☐	☐	☐	Helped us to implement successfully

Please briefly describe the social architecture of your service setting (i.e. size, age, level of expertise):

Practitioner turnover

	-2	-1	0	1	2	
Hindered our attempts to implement successfully	☐	☐	☐	☐	☐	Helped us to implement successfully

Leadership turnover

	-2	-1	0	1	2

Hindered our attempts to implement successfully	◻	◻	◻	◻	◻	Helped us to implement successfully

Division of labour among DBT practitioners

	-2	-1	0	1	2	
Hindered our attempts to implement successfully	◻	◻	◻	◻	◻	Helped us to implement successfully

Decision-making autonomy within your service

	-2	-1	0	1	2	
Hindered our attempts to implement successfully	◻	◻	◻	◻	◻	Helped us to implement successfully

Availability of DBT networks

	-2	-1	0	1	2	
Hindered our attempts to implement successfully	◻	◻	◻	◻	◻	Helped us to implement successfully

Feedback or other communication about DBT outcomes across the organisation

	-2	-1	0	1	2	
Hindered our attempts to implement successfully	◻	◻	◻	◻	◻	Helped us to implement successfully

Compatability of DBT with organisational values and goals

	-2	-1	0	1	2	
Hindered our attempts to implement successfully	◻	◻	◻	◻	◻	Helped us to implement successfully

The absorptive capacity for change within your service

	-2	-1	0	1	2	
Hindered our attempts to implement successfully	◻	◻	◻	◻	◻	Helped us to implement successfully

Shared willingness to implement DBT among DBT trained clincians

	-2	-1	0	1	2	

Hindered our attempts to implement successfully	☐	☐	☐	☐	☐	Helped us to implement successfully

Leadership engagement with DBT

	-2	-1	0	1	2	
Hindered our attempts to implement successfully	☐	☐	☐	☐	☐	Helped us to implement successfully

Availability of resources

	-2	-1	0	1	2	
Hindered our attempts to implement successfully	☐	☐	☐	☐	☐	Helped us to implement successfully

Shared perception of the importance of implementing DBT in your service

	-2	-1	0	1	2	

Hindered our attempts to implement successfully	☐	☐	☐	☐	☐	Helped us to implement successfully

***Learning climate within your service** (e.g. the extent to which individuals feel psychologically safe to try new methods and where sufficient time and space is provided to do so).*

	-2	-1	0	1	2	
Hindered our attempts to implement successfully	☐	☐	☐	☐	☐	Helped us to implement successfully

Would you like to expand further on any of the responses you have provided on this page?

Characteristics of Individuals

Practitioner attitudes towards DBT

	-2	-1	0	1	2	
Hindered our attempts to implement successfully	☐	☐	☐	☐	☐	Helped us to implement successfully

Skills of DBT practitioners

	-2	-1	0	1	2	
Hindered our attempts to implement successfully	☐	☐	☐	☐	☐	Helped us to implement successfully

Practitioner readiness for DBT

	-2	-1	0	1	2	
Hindered our attempts to implement successfully	☐	☐	☐	☐	☐	Helped us to implement successfully

Would you like to expand further on any of the repsonses you have provided on this page?

Implementation Process

Level of planning required for implementation tasks

	-2	-1	0	1	2	
Hindered our attempts to implement successfully	☐	☐	☐	☐	☐	Helped us to implement successfully

Selection process of DBT practitioners

	-2	-1	0	1	2	
Worked against successful implementation	☐	☐	☐	☐	☐	Worked towards successful implementatior

Appointment of DBT leader(s)

	-2	-1	0	1	2	
Hindered our attempts to implement successfully	☐	☐	☐	☐	☐	Helped us to implement successfully

Existence of DBT champion(s)

	-2	-1	0	1	2	

Hindered our attempts to implement successfully	☐	☐	☐	☐	☐	Helped us to implement successfully

Influence of external change events

	-2	-1	0	1	2	
Hindered our attempts to implement successfully	☐	☐	☐	☐	☐	Helped us to implement successfully

Execution of implementation plan

	-2	-1	0	1	2	
Hindered our attempts to implement successfully	☐	☐	☐	☐	☐	Helped us to implement successfully

Evaluation and feedback of implementation efforts

	-2	-1	0	1	2	
Hindered our attempts to implement successfully	☐	☐	☐	☐	☐	Helped us to implement successfully

Would you like to expand further on any of the responses you have provided on this page?

Finish

Please enter your email address below:

Would you like to receive a short summary of the results of the study prior to them being made available to the public?

- Yes
- No

Thank you!

Thank you for taking the time to complete the survey.
